# Identification of ordinal relations and alternative suborders within high-dimensional molecular data

**DOI:** 10.3389/fbinf.2025.1665892

**Published:** 2025-11-03

**Authors:** Ana Stolnicu, Peter Eckhardt-Bellmann, Angelika M. R. Kestler, Hans A. Kestler

**Affiliations:** 1 Institute of Medical Systems Biology, Ulm University, Ulm, Germany; 2 Department of Internal Medicine I, Ulm University Hospital, Ulm, Germany; 3 Leibniz Institute on Aging – Fritz Lipmann Institute, Jena, Germany

**Keywords:** alternative progression patterns, classifier cascades, directed threshold classifiers, ordinal classification, high-throughput data

## Abstract

**Introduction:**

Numerous biological systems exhibit ordinal connections between categories. Developmental and time-series information inherently depict sequences like “early,” “intermediate,” and “late” phases, showing that these specific processes follow a progression. Ordinal classification techniques are often applied in biological and medical contexts, ranging from the evaluation of pain intensity, to the detection of evolving diseases, such as cancer. These ranking systems may assist clinicians in establishing diagnoses and developing tailored treatment plans. For instance, tumor staging might guide early detection strategies and targeted therapies, improving patient outcomes. However, applying ordinal classification to biological data presents considerable challenges. In addition to their high dimensionality, these datasets can be highly heterogeneous, often reflecting branching processes that occur simultaneously during progression. Factors such as intratumoral diversity, asynchronous progress, and context-specific signaling activity may interfere with the identification of such alternative development routes.

**Methods:**

To address these challenges, we propose a framework for uncovering ordinal relationships within molecular data. Specifically, directed threshold classifiers are introduced as base learners for ordinal classifier cascades, enabling the detection of both total and partial orderings between molecular states.

**Results:**

This approach preserves the inherent ordinal structure by projecting high-dimensional data onto one single dimension while simultaneously decreasing complexity. Additionally, the distinct features of the resulting thresholds allow the prediction of potential alternative paths among the suborders.

## 1 Introduction

Various physiological processes and health conditions naturally follow an ordinal arrangement, in which stages progress hierarchically (Prigogine and Nicolis, 1971). Organizing disease phases into meaningful semantic groups can be a valuable predictive tool in clinical practice (Lee et al., 2004). In oncology, tumor classification aids in prognosis and treatment strategies, guiding the choice of interventions and targeted therapies based on predicted tumor behavior (Beadsmoore and Screaton, 2003; Forner et al., 2014; Cortés et al., 2014). Similarly, categorizing the stages of neurodegenerative disorders, such as Alzheimer’s disease (Sperling et al., 2011; Davis et al., 2018; Tahami Monfared et al., 2022) might facilitate prompt therapeutic decisions and patient monitoring (Scharre, 2019). Pain classification adheres to comparable principles, where pain intensity reported by patients can be arranged into numerical or categorical scales (Hadjistavropoulos and Craig, 2002; Haefeli and Elfering, 2006). These may also be used in anesthesiology and pain management to guide treatment suitability (Breivik et al., 2008). Despite its clinical utility, ordinal classification in biological and medical data presents considerable computational challenges. Molecular datasets, such as gene expression profiles, are not only high-dimensional, consisting of thousands of interrelated features, but also may encode multiple, potentially parallel biological processes, each with its own progression dynamics (Brody, 2009; Wu et al., 2019; Yang et al., 2022; Gerlinger et al., 2012). Additionally, high-throughput data often suffer from noise introduced by experimental variability, batch effects, and underlying biological diversity, which can hinder ordinal relationships and complicate model training (Tu et al., 2002; Goh et al., 2017). Moreover, numerous biological processes lack clear stage transitions, exhibiting overlapped molecular signatures and divergent trajectories, leading to ambiguous classification boundaries and partial ordering of states (Seoane and De Mattos-Arruda, 2014). Further suggesting that these mechanisms could potentially evolve through various parallel pathways (Olschwang et al., 1997; Traverso et al., 2002).

The proposed architecture is tailored for the detection of ordinal structures within one-dimensional data, derived from high-throughput datasets. The categories, i.e., classes, are delineated by thresholds that partition the input space into distinct intervals. An essential aspect of this method is the ability to recreate potential alternative ordinal trajectories from the resulting suborders. This can be accomplished based on the properties of the decision boundaries. As a result, the model can provide a distinct benefit in scenarios in which the ordinal structure may not be strictly predefined, allowing for more nuanced and adaptable classification decisions. However, note that our introduced architecture is designed for the detection of ordinal structures and (parallel) substructures, not for the classification process at hand.

## 2 Related work

Ordinal classification is a type of supervised learning in which the classes exhibit an intrinsic order that does not necessarily adhere to specific numerical intervals (Frank and Hall, 2001). In contrast to conventional ordinal classification approaches, which typically assume a fixed class order and often fail to capture the optimal ordinal correlations between classes, ordinal classifier cascades (OCCs) (Lattke et al., 2015) decompose the task into a series of simplified binary classification problems. In this framework, a cascade of classifiers is used, where each classifier determines whether a given instance belongs to a specific category or a higher-ranked one. The cascade approach evaluates samples sequentially, attributing a label according to the first classifier that provides a confident prediction. This structure not only streamlines the classification problem at each stage, but also allows the exploration of potential class sequences. In this context, the CASCADES algorithm (Lausser et al., 2019) extends the sequential framework by improving its efficiency, replacing the exhaustive search with exploratory screening of candidate orders. To handle the computational complexity of this search, it employs early rejection criteria based on class-wise sensitivity limits, discarding underperforming cascades prior to complete training. Additionally, binary classifiers in the cascade are trained to distinguish between a class and its successor, enabling pairwise trained classifiers to be stored and reused for different input orders, thus decreasing runtime and minimizing redundant computations. Because the algorithm is independent of the classifier type, allowing the integration of any suitable binary training method, this approach enhances both efficiency and flexibility. Finally, it produces a set of candidate cascades that satisfy the established performance criteria, which can be further evaluated for ensemble integration or downstream model selection.

Formally, in the context of ordinal classification, we are given a set of N samples, $D={\left\{\left(x_{k},y_{k}\right)\right\}}_{k=1}^{N}$ , where $x_{k}\in X$ denotes the feature vector of the k -th sample and $y_{k}\in L$ indicates its associated label. Here, $X\subseteq R^{d}$ is the feature space and $L=\left\{l_{1},l_{2},\ldots ,l_{\left|L\right|}\right\}$ corresponds to the finite set of class labels. The objective is to predict the label for each sample k taking into account its feature vector. Thus, a binary classifier, $c_{\left(i,i+1\right)}$ , of an OCCs ensemble, $\varepsilon_{C}$ , is trained to differentiate between samples belonging to adjacent classes, $l_{i}$ and $l_{i+1}$ , in the given semantic order, $l_{1}≺l_{2}≺\cdots ≺l_{\left|L\right|}$ , as:

$$\varepsilon_{C}=\left\{c_{\left(i,i+1\right)}:X\mapsto \left\{l_{i},l_{i+1}\right\}|i=1,\ldots ,|L|-1\right\}.$$ (1)

The index i designates the position of the classes in the given order. Throughout the classification procedure, every $x_{k}$ is evaluated by the sequence of classifiers arranged according to the order under investigation, that is, if the input order is $o=l_{1}≺l_{2}≺\cdots ≺l_{\left|L\right|}$ , the classifiers are organized as $\left\{c_{\left(1,2\right)},c_{\left(2,3\right)},\ldots ,c_{\left(\left|L|-1,|L\right|\right)}\right\}$ . For a sample k , if a classifier $c_{\left(i,i+1\right)}\left(x_{k}\right)$ generates a positive prediction for the first label, $l_{i}$ , the corresponding label will be assigned to $x_{k}$ , and the cascade ends. Otherwise, the sample is passed to the next classifier in the sequence, continuing the process until the final classifier $c_{\left(\left|L|-1,|L\right|\right)}$ is reached, in which case, if the second label is predicted, then the predicted label $y_{k}^{\prime }$ is equal to $l_{\left|L\right|}$ , as defined in Equation 2:

$$y_{k}^{\prime }=\left\{\begin{array}{cc} l_{j},\; & \text{where}\;j=\min\left\{i\in \left\{1,\ldots ,|L|-1\right\}\;|\;c_{\left(i,i+1\right)}\left(x_{k}\right)=l_{i}\right\},\\ l_{\left|L\right|},\; & \text{if}\;c_{\left(i,i+1\right)}\left(x_{k}\right)=l_{i+1}\;\forall i<|L|. \end{array}\right.$$ (2)

**FIGURE 1:**
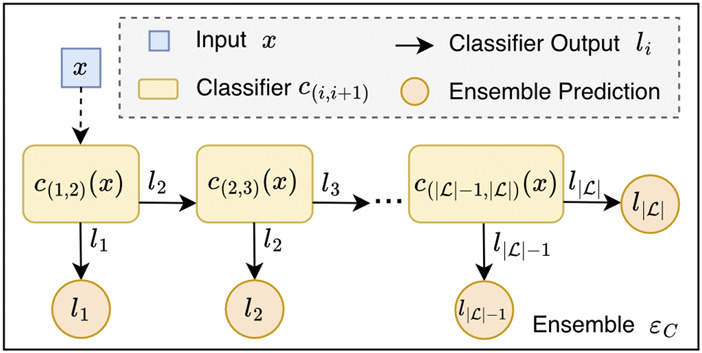
Ordinal classifier cascade (OCC) ensemble. The OCC architecture consists of |L|−1 binary classifiers $c_{\left(i,i+1\right)}$ that can either predict label $l_{i}$ or label $l_{i+1}$ . If the *greater* class $\left(l_{i+1}\right)$ is predicted, the input is passed to the next classifier in the sequence. Otherwise, if the *lower* class $\left(l_{i}\right)$ is predicted by $c_{\left(i,i+1\right)}$ , then this output is taken as the ensemble’s final prediction for input x . The last classifier in the sequence, $c_{\left(\left|L|-1,|L\right|\right)}$ , cannot further pass input x , and therefore, once reached, always provides the ensemble’s final output, by predicting either $l_{|L|-1}$ or $l_{\left|L\right|}$ . An OCC ensemble is defined by its set of classifiers, as in Equation 1.

In order to guide the selection of the most effective cascades, the class-wise sensitivity serves as primary efficiency criterion for the classifiers. An example of an OCC architecture is depicted in Figure 1.

Bellmann and Schwenker (2020) proposed another approach for the detection of ordinal class structures, in which it is not necessary to explicitly evaluate all possible class orderings. The idea is to determine the performance (resubstitution accuracy) of linear Support Vector Machines (SVMs) (Vapnik, 2000) for each class pair, i.e., |L|⋅(|L|−1)/2 binary subtasks. The resulting performance values, $a_{i,j}$ , imply how well the classes, $\left\{l_{i},l_{j}\right\}$ , can be separated from each other. As the next step, the values $a_{i,j}$ are combined into |L| symmetric matrices A , $A={\left(a_{i,j}\right)}_{i,j=1}^{\left|L\right|}$ , with different arrangements of the row (and column) elements. While the symmetry of each A is obtained by definition, due to $a_{i,j}=a_{j,i}$ , for all i≠j , the authors defined $a_{i,i}≔0$ , ∀i=1,…,|L| . An ordinal class structure is found if and only if there exist exactly two matrices A for which the row (and column) entries are monotonously decreasing towards the diagonal elements. From the symmetry characteristic, it follows that each ordinal structure is found together with its reverse order.

Bellmann and Schwenker (2020) further extended their work in (Bellmann et al., 2022). They generalized their working definition of ordinal classification tasks by introducing a theoretical framework which makes it possible to detect ordinal class structures without utilizing any classification model. As an example, they proposed using a multidimensional adaptation of Fisher’s discriminant ratio (Fisher, 1936). Using their framework, they proved that, in general, 3-class classification problems can be regarded as ordinal classification tasks consisting of two edge classes and a class identified as the central one. Note that the authors reduced the detection complexity from evaluating all possible class orderings, |L|! evaluations, to only |L|⋅(|L|−1)/2 . However, they did not discuss the potential for detecting substructures, a useful property that was elaborated by Lausser et al. (2020) based on the CASCADES algorithm. In contrast to the methods discussed above, in our current approach, the mining for ordinal suborders is not conducted in the provided, and often high-dimensional, feature space, but in combination with the one-dimensional real space. Moreover, with our approach presented in this work, we are able to identify alternate progressions.

## 3 Materials and methods

### 3.1 Directed threshold classifiers

The purpose of the Directed Threshold Classifiers (DTCs) introduced in this work is to recognize ordinal relations within univariate data X⊆R . A DTC $f_{\tau }:X\to \left\{l_{i},l_{j}\right\}$ , defined by a threshold τ∈R , is built to differentiate between two distinct categories $l_{i},l_{j}$ :

$$f_{\tau }\left(x\right)=\left\{\begin{array}{cc} l_{j},\; & if\;x\geq \tau ,\\ l_{i},\; & otherwise. \end{array}\right.$$ (3)

The threshold τ divides the input space into two decision areas, in which all elements belonging to class $l_{j}$ , which have values greater than τ , are assigned on the right side, whereas instances of class $l_{i}$ , with values below τ fall into the region on the left side, as shown in Equation 3. A set of DTCs can be organized sequentially according to a specified input order $o=l_{1}≺\cdots ≺l_{\left|L\right|}$ to be further applied as base classifiers within the OCCs framework. The samples being examined are assumed to be arranged along a one-dimensional axis, and the thresholds, corresponding to specific points, are constrained to follow a strictly increasing order on the same line, $\tau_{1}<\ldots <\tau_{|L|-1}$ . This guarantees that the decision regions form contiguous segments within the space, leading to a connected and non-overlapping partitioning of the domain that mirrors a consistent progression aligned with the ordinal nature of the targeted labels. Moreover, the non-intersecting characteristic of the regions inherently creates parallel decision boundaries, as each one is orthogonal to the axis of progression. For the computation of the one-dimensional thresholds, we apply linear SVM models, making use of their margin maximization characteristic.

### 3.2 Data transformation to one dimension

As univariate data rarely appear in real-world scenarios, the first step of the method involves dimension reduction, for which supervised and non-supervised techniques exist. Principal components analysis (PCA) (Kambhatla and Leen, 1997), Linear discriminant analysis (LDA) (Fisher, 1936), t-distributed stochastic neighbor embedding (t-SNE) (Van der Maaten and Hinton, 2008), and uniform manifold approximation and projection (UMAP) (McInnes and Healy, 2018) are just a few of the numerous applicable methods that can be used. In this section, we provide a different strategy tailored to meet the specific objective of our study. The process is summed up in the following main steps: From the available category set we select a pair of classes, $\left(l_{i},l_{j}\right)$ , to which we apply a linear binary classifier. The data points are then projected onto the orthogonal hyperplane of the resulting linear model. For this binary linear classification, we employed SVMs, in which the data were streamlined to a one-dimensional form by mapping the points using the normal vector.

Note that we prioritized SVM models for the mapping of the high-dimensional data onto one dimension for the following main reasons. First, SVM models are supervised, i.e., classes play an important role during projection. Second, SVMs are deterministic, ensuring reproducibility. In addition, SVM models maximize the margin between the classes of the chosen projection class pair, which we consider to be important when mining for ordinal structures in the one-dimensional space. However, users of our introduced approach can replace the SVM-based projection by any projection of their preferred choice.

Given that the selection of the initial data mapping most likely affects the direction of the DTCs during the overall screening process, a key aspect to take into account is the choice of this class pair. Despite appearing trivial, it is important to notice that the two classes are maintained apart from each other in the classification process. Consequently, the resulting projection is likely to highlight distinctions between these selected classes, potentially overlooking variations or correlations in the other classes. In the experiments reported in this work, we examined every possible pairwise combination. We observed that using the two least related categories in the developmental process described by the dataset, generally produced the most consistent results.

### 3.3 Alternative progressions

In the cascaded system, both total orders and potential suborders can be identified. When partial configurations emerge, it may be particularly valuable to investigate whether they reflect alternative advancements of the same underlying progression. In this context, the afore described properties of the thresholds can help uncover and characterize competing developmental paths. For suborders to be considered as potential parallel trajectories of the same process, they must share a subset of thresholds. In the following, we formally define the criteria that determine when a threshold qualifies as shared between suborders. Let $L=\left\{l_{1},\ldots ,l_{\left|L\right|}\right\}$ represent a finite collection of class labels for which no global order is determined. Assume that two suborders, o⊂L and $o^{\prime }\subset L$ , can be recognized so that each defines a valid ordinal sequence. Suppose that a classifier system exists according to which the associated decision threshold is identified with minimal class-wise sensitivity, sens=1 , for every category within the respective suborders (i.e., all class instances are correctly classified). The threshold sets obtained for the suborders o and $o^{\prime }$ can be denoted as $\tau_{o}=\left\{\tau_{1},\ldots ,\tau_{k}\right\}$ and $\tau_{o^{\prime }}=\left\{\tau_{1}^{\prime },\ldots ,\tau_{m}^{\prime }\right\}$ , respectively. We are interested in identifying whether a threshold equivalence relation $\tau_{i}\equiv \tau_{j}^{\prime }$ , with $\tau_{i}\in \tau_{o}$ and $\tau_{j}^{\prime }\in \tau_{o^{\prime }}$ , can be established. Two thresholds are deemed equivalent if they induce identical separation boundaries in regions in which two distinct classes have the same adjacent class in their respective suborders. A threshold can be left-shared or right-shared, depending on whether the common neighboring class is on the left or on the right side of the two categories, detailed in Equations 4–10. Formally, given the classes $l_{a}\in o\backslash o^{\prime }$ , $l_{b}\in o^{\prime }\backslash o$ and $l_{l}\in o\cap o^{\prime }$ , if the subsequent inequalities occur,

$$X_{l_{l}}<\tau_{i}\leq X_{l_{a}}\text{ and }$$ (4)

$$X_{l_{l}}<\tau_{j}^{\prime }\leq X_{l_{b}},$$ (5)

where $X_{l_{i}}$ represents the set of feature values associated with class $l_{i}$ , then $\tau_{i}\equiv \tau_{j}^{\prime }$ . Moreover, a threshold $\tau_{ls}$ exists such that $\tau_{i},\tau_{j}^{\prime }\mapsto \tau_{ls}$ , where $\tau_{ls}$ represents the left-shared threshold between the respective class transitions. Similarly, if the following inequalities arise,

$$X_{l_{a}}<\tau_{i}\leq X_{l_{r}}\text{ and }$$ (6)

$$X_{l_{b}}<\tau_{j}^{\prime }\leq X_{l_{r}},$$ (7)

then $\tau_{i}\equiv \tau_{j}^{\prime }$ , and a threshold $\tau_{rs}$ exists, such that $\tau_{i},\tau_{j}^{\prime }\mapsto \tau_{rs}$ represents the right-shared threshold of $l_{a}$ and $l_{b}$ . It follows that $\tau_{ls}$ will be situated between $l_{l}$ and the minimum among $l_{a}$ and $l_{b}$ , whereas, $\tau_{rs}$ has to be greater than the maximum of $l_{a}$ and $l_{b}$ and less than $l_{r}$ :

$$X_{l_{l}}<\tau_{ls}\leq \min\left\{X_{l_{a}},X_{l_{b}}\right\},$$ (8)

$$\max\left\{X_{l_{a}},X_{l_{b}}\right\}<\tau_{rs}\leq X_{l_{r}}.$$ (9)

In a wider framework, in which incorrect sample classifications are allowed with a misclassification rate of θ=1−sens , with sens∈[0.5,1] , θ can be incorporated to define the thresholds between each pair of adjacent classes $l_{i}$ and $l_{i+1}$ as:

$$\tau \in \left[X_{l_{i}}+\theta \cdot \left(X_{l_{i+1}}-X_{l_{i}}\right),X_{l_{i+1}}-\theta \cdot \left(X_{l_{i+1}}-X_{l_{i}}\right)\right].$$ (10)

Two suborders are required to have a common minimal class sensitivity for each involved class to qualify as viable alternatives, thus left- and right-shared thresholds can be adapted to account for the same amount of misclassifications as outlined below:

$$\begin{array}{ll} \tau_{ls} & >X_{l_{l}}+\theta \cdot \min\left\{X_{l_{a}}-X_{l_{l}},\;X_{l_{b}}-X_{l_{l}}\right\},\\ \tau_{ls} & \leq \min\left\{X_{l_{a}}-\theta \cdot \left(X_{l_{a}}-X_{l_{l}}\right),\;X_{l_{b}}-\theta \cdot \left(X_{l_{b}}-X_{l_{l}}\right)\right\},\\ \tau_{rs} & >\max\left\{X_{l_{a}}-\theta \cdot \left(X_{l_{a}}-X_{l_{l}}\right),\;X_{l_{b}}-\theta \cdot \left(X_{l_{b}}-X_{l_{l}}\right)\right\},\\ \tau_{rs} & \leq X_{l_{l}}+\theta \cdot \max\left\{X_{l_{a}}-X_{l_{l}},;\;X_{l_{b}}-X_{l_{l}}\right\}. \end{array}$$

**FIGURE 2:**
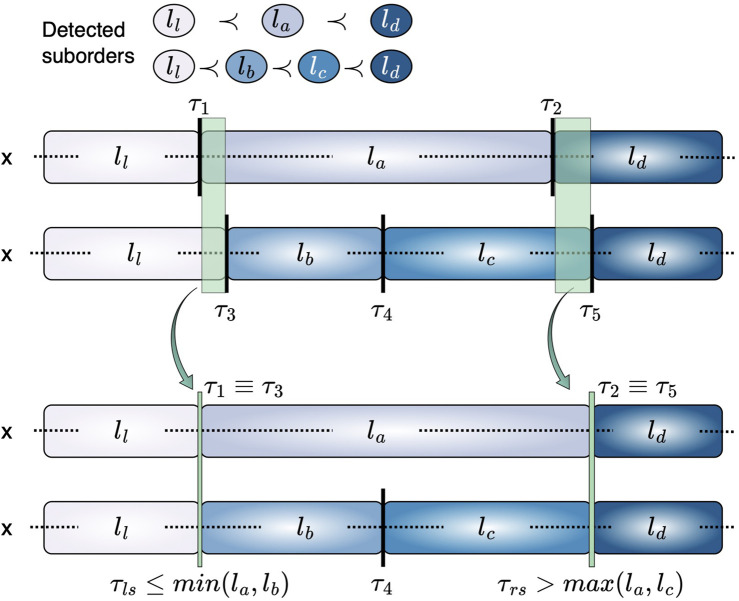
Representation of equivalent thresholds across suborders. For suborders $l_{l}≺l_{a}≺l_{d}$ and $l_{l}≺l_{b}≺l_{c}≺l_{d}$ , $\tau_{1}$ and $\tau_{3}$ share $l_{l}$ on the left, similarly $\tau_{2}$ and $\tau_{5}$ share $l_{d}$ on the right. This characteristic allows to consider $\tau_{1}$ and $\tau_{3}$ , as well as $\tau_{2}$ and $\tau_{5}$ , as equivalent, enabling the arrangement of the two suborders as alternatives of the same phenomenon.

This ensures that the decision boundaries retain a consistent level of ambiguity across class transitions. The concept of shared thresholds is illustrated in Figure 2.

**FIGURE 3:**
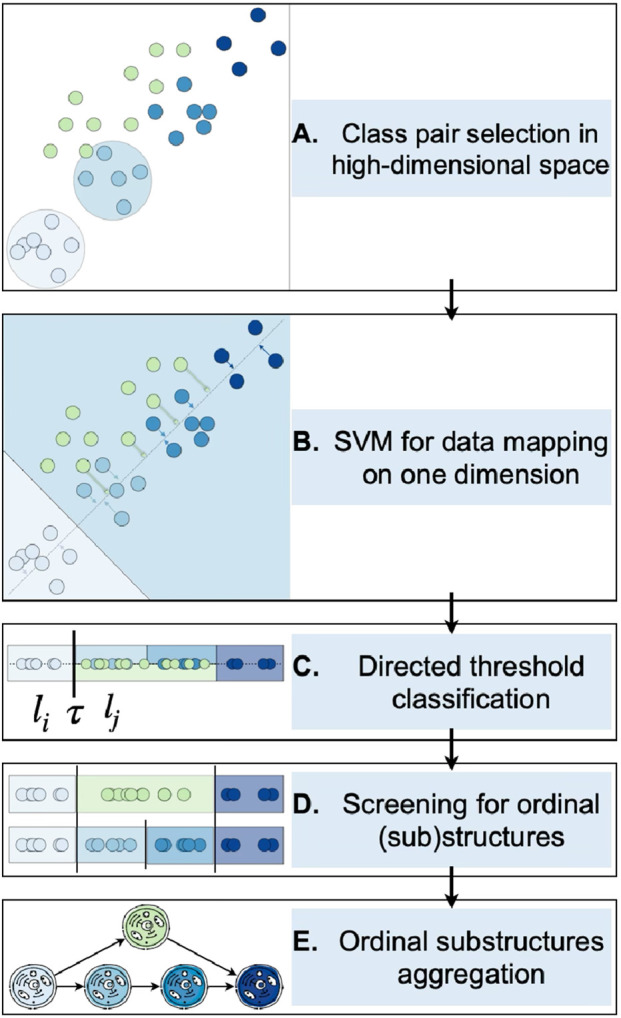
Depiction of the entire process for identifying ordinal structures in molecular high-throughput data. Steps A to B illustrate the data projection, beginning with the selection of a pair of classes **(A)** on which a binary linear classifier is utilized, followed by the projection of the data onto the boundary’s perpendicular **(B)**. Consequently, the directed threshold classifiers are applied on the one-attribute observations **(C)**. Ordinal patterns are found using an extensive screening procedure by means of ordinal classifier cascades **(D)** which are subsequently analyzed to ascertain potential alternative trajectories **(E)**.

A visual representation of the designed procedure is provided in Figure 3, beginning with the data projection (A–B), followed by the application of DTCs and the screening procedure to extract ordinal substructures (C–D), and concluding with their aggregation for the retrieval of potential alternative structures (E).

### 3.4 Reversed orders

Another feature of this approach lies in the implicit retrieval of inverted suborders. More precisely, for a specific class pair $\left(l_{i},l_{j}\right)$ , if the sequence $o=l_{1}≺\cdots ≺l_{\left|L\right|}$ is retrieved, applying the inverse combination, i.e., $\left(l_{j},l_{i}\right)$ , for the data transformation yields the reversed order $o^{\prime }=l_{\left|L\right|}≺\cdots ≺l_{1}$ . This behavior arises from the fact that switching the class pairs results in a mapping transformation that mirrors the original structure, thereby naturally producing the converse sequence without additional interventions. By definition, this means that reversed orders are mathematical artifacts. Whether the reversal is biologically meaningful depends on the classification task at hand and has to be discussed for each case individually.

### 3.5 Analyzed datasets

The method was initially evaluated using synthetic data comprising 10 distinct categories, $l_{0},\ldots ,l_{9}$ , each containing 100 samples described by two features, which were further reduced to one dimension using the technique introduced in Section 3.2.

To validate our approach, we used two publicly available developmental datasets from the Gene Expression Omnibus (GEO) (Barrett et al., 2012). The expression measurements of 4028 genes of *Drosophila melanogaster* (*D. melanogaster*) (Arbeitman et al., 2002) (included in GEO accession number: GSE4347) were taken at various stages of the fruit fly’s life cycle. The developmental phases can be arranged as embryo≺larva≺pupa≺adult , with 31, 10, 18 and 8 samples in each category, respectively. The second dataset is composed by pineal glands gene expression profiles collected at five distinct time periods of the zebrafish’s (*D. rerio*) maturation process (Toyama et al., 2009) (GEO accession number: GSE13371). They cover three embryonic (3 days, 5 days, and 10 days) and two adult time points (3 months, 1–2 years). The first group consists of 14, 14, and 15 samples, respectively, whereas the second group comprises 12 and 14 samples, respectively.

Furthermore, we used two tumor datasets to test our methodology. The pancreatic ductal adenocarcinoma (PDAC) (Buchholz et al., 2005) which includes 21521 gene expression profiles from human microdissected cells, with 38 samples split into 5 classes: normal ductal cells (6 samples), three intermediate pancreatic intraepithelial neoplasia (PanIN), PanIN-1 (6 samples), PanIN-2 (8 samples) and PanIN-3 (10 samples), as well as the metastatic stage (PDAC) (8 samples). This process is assumed to develop according to the sequence normal ≺ PanIN-1 ≺ PanIN-2 ≺ PanIN-3 ≺ PDAC. The pancreatic neuroendocrine tumors (PanNET) (Sadanandam et al., 2015) (GEO accession number: GSE73514) comprise 35511 mutational profiles from the RIP1 TAG2 mouse model, containing 22 samples organized into 6 categories: 3 samples for each normal mature β -cells (NM), hyperplastic islet (HI), angiogenic islet (AI) and liver metastasis (MET), and 5 samples for tumor islet (TI) and met like primary (MLP), each. The assumed progression is NM ≺ HI ≺ AI ≺ TI ≺ MLP ≺ MET.

For all non-synthetic datasets analyzed in this work, we utilized the normalized versions of the samples provided by the original authors to ensure reproducibility. Details of the normalization procedures can be found in the respective dataset publications (Arbeitman et al., 2002; Toyama et al., 2009; Buchholz et al., 2005; Sadanandam et al., 2015). For the zebrafish dataset (Toyama et al., 2009) we additionally applied a $log_{2}$ transformation to stabilize variance and diminish asymmetry.

## 4 Results

### 4.1 Synthetic data simulations

**FIGURE 4:**
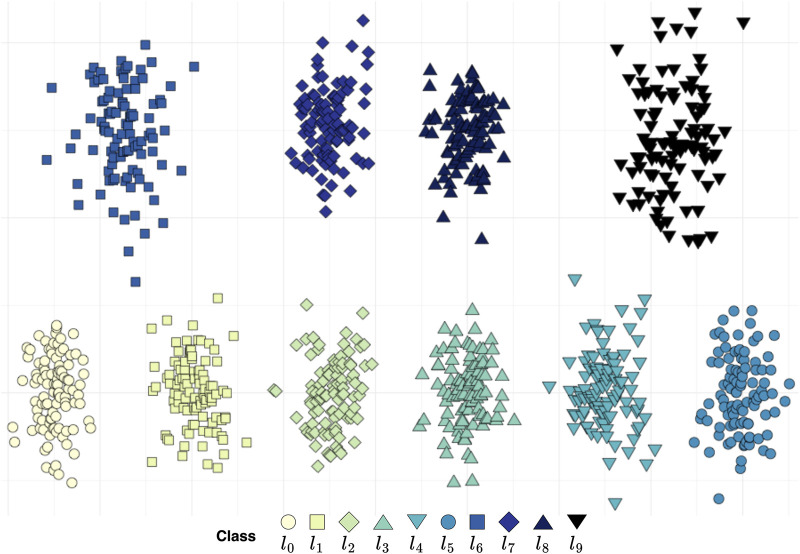
Synthetic two-dimensional data. Within the ten classes, no total order can be found, yet four suborders of length six are present: $\left(l_{0}≺\cdots ≺l_{5}\right)$ , $\left(l_{0}≺l_{1}≺l_{2}≺l_{8}≺l_{4}≺l_{5}\right)$ , $\left(l_{0}≺l_{1}≺l_{7}≺l_{3}≺l_{4}≺l_{5}\right)$ and $\left(l_{0}≺l_{1}≺l_{7}≺l_{8}≺l_{4}≺l_{5}\right)$ . In addition, 8 subsequences of length five can be likewise identified, resembling the previous ones where sequences $\left(l_{0}≺l_{1}\right)$ and $\left(l_{4}≺l_{5}\right)$ are replaced by classes $l_{6}$ and $l_{9}$ , respectively.

**FIGURE 5:**
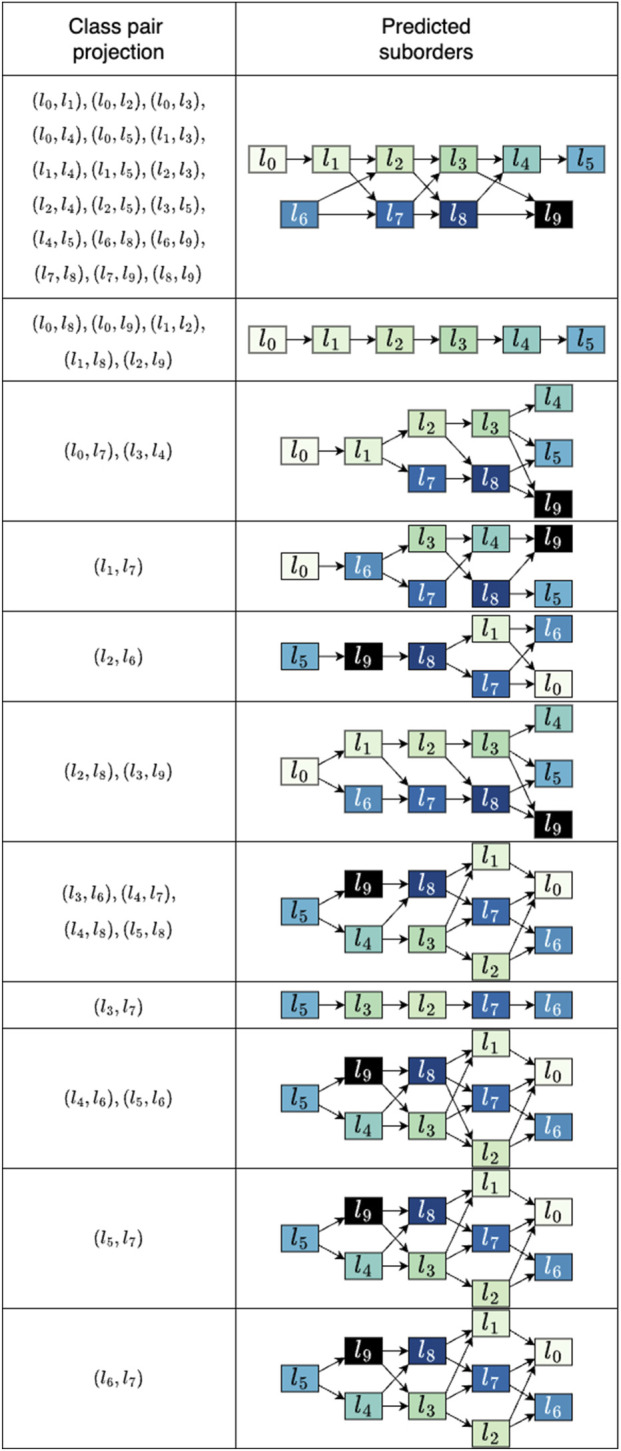
Predicted suborders for the synthetic data. For every outcome the corresponding pairs of classes used to project the data are listed. The resulting suborders are depicted as aggregated graphs where overlapping classes can be seen as alternatives. Only results that produced sequences of lengths six and five are shown. The average runtime for suborder screening across the 45 projections was 0.10 s.

Upon considering either dimension of the simulated data, no ordinal arrangement encompassing all classes can be discerned with minimal class sensitivity of 1, as illustrated in Figure 4. To investigate the impact of the data projection on the final outcome, we employed all pair combinations of the categories which is the design of the linear decision boundary. The class pairings that returned orders of length six or five are shown in Figure 5. The suborders are illustrated in a concise graph where overlapping categories, or groups, are shown layered atop each other. For example, in the first graph, the sequence $\left(l_{0}≺l_{1}\right)$ extends alongside class $l_{6}$ , likewise $l_{2}$ overlaps with $l_{7}$ , $l_{3}$ with $l_{8}$ , and $\left(l_{4}≺l_{5}\right)$ with $l_{9}$ .

It can be seen that the suborders $\left(l_{0}≺l_{1}≺l_{2}≺l_{3}≺l_{4}≺l_{5}\right)$ and $\left(l_{6}≺l_{7}≺l_{8}≺l_{9}\right)$ appear in the outcomes obtained from various combinations. The majority consists of class couples that incorporate categories from the same suborder, for instance $l_{0}$ paired with any other class among $\left\{l_{1},\ldots ,l_{5}\right\}$ or $l_{9}$ with any class from $\left\{l_{6},l_{7},l_{8}\right\}$ . Six out of 45 combinations, namely $\left(l_{0},l_{6}\right)$ , $\left(l_{1},l_{6}\right)$ , $\left(l_{2},l_{7}\right)$ , $\left(l_{3},l_{8}\right)$ , $\left(l_{4},l_{9}\right)$ and $\left(l_{5},l_{9}\right)$ , produced suborders with lengths less than four and are excluded from the shown results. Particularly poor were the sequences obtained from the data mapping of pair $\left(l_{2},l_{7}\right)$ , in which only orders of length two were identified.

### 4.2 Empirical datasets

**FIGURE 6:**
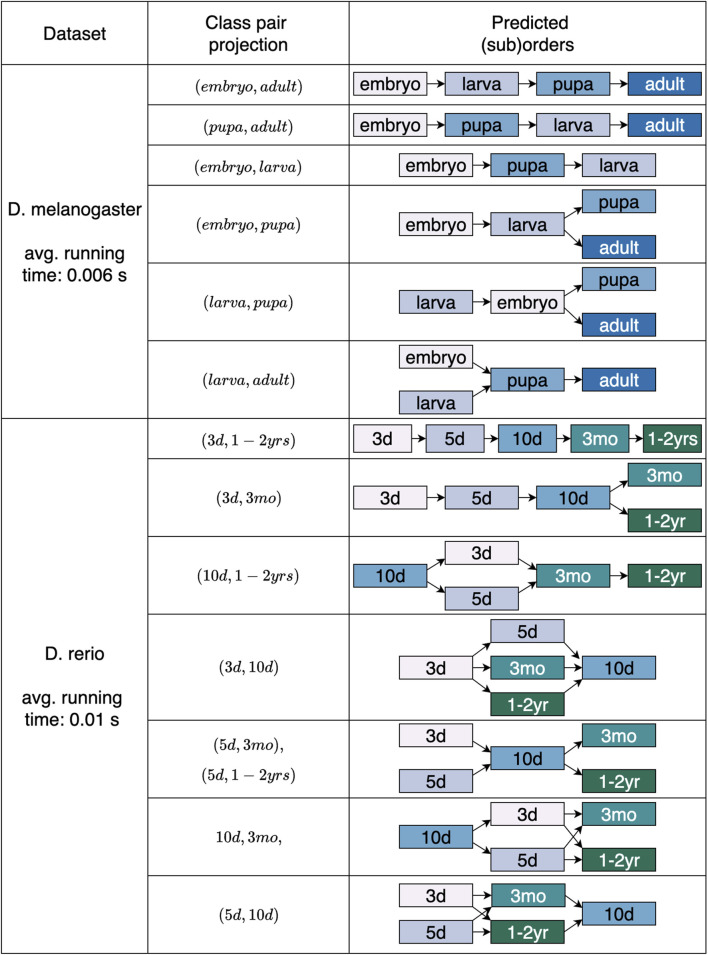
Predicted overall and partial sequences for the two developmental datasets. The projecting class pairs that returned orders either matching the length of the expected order or one element shorter (assumed length − 1) are provided for both *Drosophila melanogaster* and *Danio rerio*. The average computation time for suborders detection in all projections is also provided.

We additionally analyzed our approach using the developmental datasets. Alongside the employed projection class pair, Figure 6 presents the outcomes of length four and three achieved for *D. melanogaster* and of lengths from five to three for *D. rerio*. After projecting the data based on class pair (embryo, adult), being the first and last stages in the maturation process, our classification strategy accurately provided the fruit fly’s development, embryo≺larva≺pupa≺adult , with a minimal sensitivity of at least 0.9 for all the classes. However, when the data was projected using (pupa,adult) , a slightly different order was obtained, with minimal class sensitivity of 0.94. The reported suborders of length three were acquired with a minimum sensitivity of 1 for each class. Similarly, the zebrafish transitions, from embryo to adult, were predicted to follow the expected sequence, with sensitivity 1 for all classes, when the class projection (3d, 1–2yrs) was used. Whereas employing different class projections, the predicted orders exhibit some discrepancies, treating nearby stages as substitutes; for instance, we frequently observe 3d overlapping with 5d, and 3mo overlapping with 1–2yrs.

**FIGURE 7:**
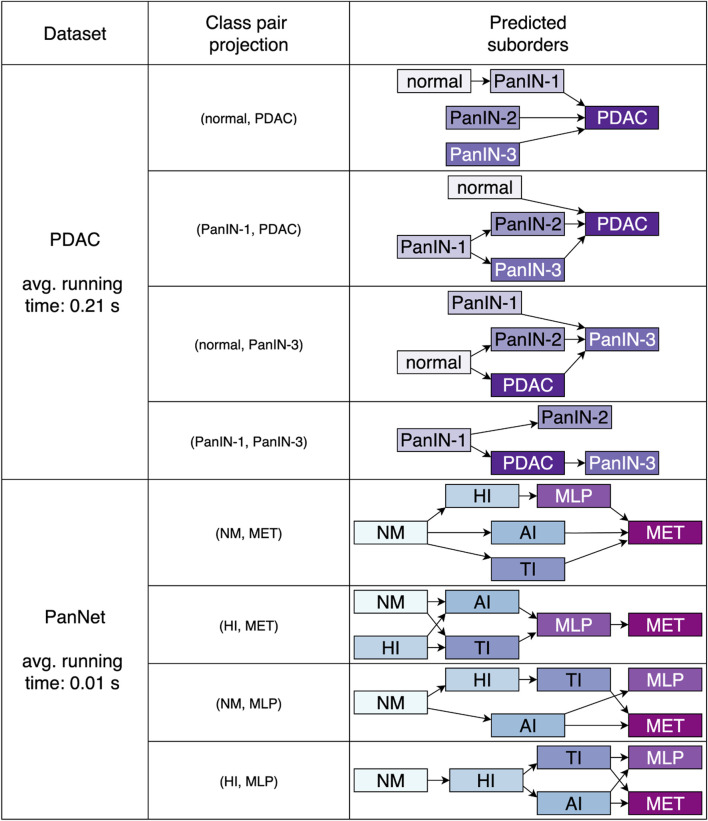
Predicted partial sequences related to pancreatic cancer, namely human PDAC and PanNET derived from the mouse model. In either case, no comprehensive orders of the entire process were predicted. However, we can observe that early phases are located in initial spots, whereas later stages are more distributed in final positions. The average runtime for the detection of suborders in all projections is also stated.

The proposed approach was further applied on the two datasets pertaining pancreatic cancer, the human PDAC and mouse PanNET. The results displayed in Figure 7 were obtained with minimal class-wise sensitivity of 1. Here, we employed projecting pairs that describe remote stages of the process under consideration. The pairs (normal, PDAC), (PanIN-1, PDAC), (normal, PanIN-3) and (PanIN-1, PanIN-3) were examined for PDAC. For PanNET we investigated (NM, MET), (HI, MET), (NM, MLP) and (HI, MLP). Each of these class combinations returned partial orders of length not greater than three for PDAC and not greater than four for PanNET.

It can be noticed that in both scenarios, no orders, comprising transitions from normal tissue to the metastatic disease, were predicted following a fully continuous or linear sequence. The observed sequences are characterized by gaps, where certain precursor lesions are noticeably absent.

### 4.3 Validation of detected structures

To validate the ordinal structures and substructures detected by our approach, we also applied the detection method introduced in (Bellmann et al., 2022). Since the method proposed by Bellmann et al. (2022) is limited to detecting total orders, we utilized it as follows. First, the complete datasets were analyzed. Subsequently, we evaluated all data subsets that contained only samples from the classes that constitute the longest substructures detected by our architecture. The evaluation led to the following outcomes.

As with our current approach, for the synthetic dataset, no total order was detected. The longest substructure consisting of six classes was confirmed, which is $l_{0}≺l_{1}≺l_{2}≺l_{3}≺l_{4}≺l_{5}$ . For *D. rerio*, the total order was confirmed, as we detected with the projection class pair (3d,1−2yrs) . For PanNET, no total order was detected. All nine suborders of length 4 were confirmed, which are depicted in Figure 7, i.e., (NM ≺ HI ≺ MLP ≺ MET), (NM ≺ AI ≺ MLP ≺ MET), (NM ≺ TI ≺ MLP ≺ MET), (HI ≺ AI ≺ MLP ≺ MET), (HI ≺ TI ≺ MLP ≺ MET), (NM ≺ HI ≺ TI ≺ MLP), (NM ≺ HI ≺ TI ≺ MET), (NM ≺ HI ≺ AI ≺ MLP), and (NM ≺ HI ≺ AI ≺ MET). For the PDAC dataset, again no total order was found and the following suborders were confirmed (cf. Figure 7): (normal ≺ PanIN-1 ≺ PDAC), (PanIN-1 ≺ PanIN-2 ≺ PDAC), and (PanIN-1 ≺ PanIN-3 ≺ PDAC). In contrast to our outcomes, for the data subset including the classes (normal, PanIN-2, PanIn-3), the approach of Bellmann et al. (2022) led to the unconventional order normal ≺ PanIN-3 ≺ PanIN-2.

The most interesting case was observed for dataset *D. melanogaster*. While we were able to detect the conventional total structure embryo ≺ larva ≺ pupa ≺ adult with the projection pair (embryo, adult), no total order was detected with the approach proposed by Bellmann et al. (2022). To further analyze this phenomenon, we additionally evaluated all data subsets including three of the four total classes. The returned detected orders of length three were (larva ≺ pupa ≺ adult), (embryo ≺ pupa ≺ adult), (embryo ≺ larva ≺ adult), and (embryo ≺ pupa ≺ larva). The last suborder seems to lead to a failed detection of a total class structure.

In summary, the comparison to the detection method introduced in (Bellmann et al., 2022) validated our detection architecture, emphasizing the benefit that our proposed approach is not limited to the analysis of total orders.

## 5 Discussion and conclusion

Uncovering ordinal correlations concealed within high-throughput data might significantly enhance our understanding of genetic alterations underlying various biological processes and assist in predicting plausible disease progression. This paper introduces a methodology to retrieve univariate representations from high-throughput datasets and further analyze them using an advanced ordinal classification framework. This approach is especially suitable when examining intricate biological mechanisms concerning, for instance, cancer progressions such as pancreatic ductal adenocarcinomas (PDACs) or pancreatic neuroendocrine tumors (PanNETs). Alongside their unpredictable non-linear progressions, these tumors often exhibit heterogeneous staging among different patients, as well as within an individual (Jones et al., 2008; Raphael et al., 2017; Witkiewicz et al., 2015; Adamo et al., 2017), indicating the likely presence of distinct alternative developmental trajectories. In order to investigate these possibilities we integrate our novel directed threshold classifiers with the existent ordinal classifier cascades. The combination of these two techniques enables the detection of underlying ordinal substructures, which can be further aggregated into partial orders to reveal potential coexisting transition routes.

The approach used for projecting the data into a single-dimensional space plays a critical role in determining the effectiveness of the identified ordinal patterns. Specifically, we observed that selecting biologically distant class pairs for the initial binary separation results in a more pronounced separability of the categories throughout the entire progression. Although the approach could benefit from additional domain expertise concerning the definition of remote stages, it still proves effective, also in its absence. One option to choose an *effective* projection class pair, without focusing on the biological meaning of the classes, is to conduct an exhaustive search over all possible class pairs and to select the two *most distant* classes, based on the provided feature space. If the biological order or possible (parallel) suborders are reflected in the provided feature space, the exhaustive search is expected to lead to a meaningful initial projection class pair. Note that, despite choosing a well-founded data transformation to one dimension, ordinal detection may be influenced by class imbalance.

The method was validated on both, synthetic and biological datasets. When applied to the artificially generated dataset, engineered to include multiple suborders, the method accurately recovered all alternative sequences. Furthermore, we successfully rebuilt known linear stage orders in developmental data from *Drosophila melanogaster* and *Danio rerio*. These preliminary findings support the suggestion that employing projection pairs describing biologically distant stages in a specific developmental process, may more effectively direct the classifier in recognizing also intermediate phases, unlike using closely related stages that might hide certain transitions. Moreover, the results also prove that the methodology is suitable for detecting overall orders encompassing all classes, as well as suborders within data that lack an underlying total order.

Predicting the staging and progression becomes more challenging when investigating oncological datasets, such as PDACs and PanNETs (Buchholz et al., 2005; Ro et al., 2013; Chan et al., 2018; Mpilla et al., 2020). In these cases, the classifiers failed to recognize a uniform and stepwise course of the diseases from the onset to the ending phase. For the human pancreatic cancer, the pancreatic intraepithelial neoplasia of degree 1 (PanIN-1) as well as dysplasias of degrees 2 (PanIN-2) and 3 (PanIN-3) appear to be followed by PDAC. This observation is consistent with the current literature characterizing pancreatic carcinomas as mostly heterogeneous tumors with a complex evolution, whereby different tumor regions can develop independently of each other (Felsenstein et al., 2018). On the cellular level, PanINs arise from neoplastic transformation of normal cells like ductal, acinar, central acinar and normal stem cells in the exocrine part of the pancreas. Various molecular changes, as well as mutations in different signaling pathways (Hedgehog, Wnt, EGF, Notch and IL-17), contribute to varying degrees to the evolution of PanIN lesions in PDAC with a key role for Notch signalling (Pian et al., 2025). This leads to the formation of many subclonal populations, supporting the hypothesis that some malignancies might not follow a single linear progression model, but rather develop through multiple, parallel evolutionary routes (Notta et al., 2017; Wu et al., 2019). Previous transcriptional profiles analyses revealed a substantial difference between lesions and malignant pancreatic tumors, with the earliest lesions resembling more closely normal tissues (Buchholz et al., 2005). This is also evident in the arrangements that result from our detection technique. The research conducted by Notta et al. (2016) revealed that approximately 65% of PDAC tumors exhibit complex chromosomal rearrangements, including chromothripsis, a phenomenon in which chromosomes massively split and rejoin in a single event (Stephens et al., 2011). Multiple tumor suppressor genes, including TP53, CDKN2A, and SMAD4, can be simultaneously inactivated by this process, leading to rapid development and spread of tumors. These findings further challenge the conventional model of incremental genetic alterations in PDAC progression, suggesting that in some cases, the disease may advance rapidly due to such genomic failures. These molecular insights also align with the sudden onset of an advanced disease and the transition of duct lesions to invasive carcinoma that have been documented in clinical settings of certain patients (Hruban et al., 1999; Al-Sukhni et al., 2012). The observation of patients undergoing yearly magnetic resonance imaging screenings revealed that although imaging could detect small pancreatic tumors and cystic lesions, some participants still developed higher stage PDAC with minimal or no prior symptoms. Pancreatic neuroendocrine tumors can be clinically differentiated into functionally active and inactive types, and further subdivided into well-differentiated and poorly differentiated subgroups. Further subtyping of this clinically heterogeneous tumor entity can be achieved by integrating molecular information that may be relevant to tumor development and progression (Shen et al., 2022). Despite the fact that PDAC and PanNETs are distinct entities, several studies highlight the role of chromatin remodeling and genomic alterations in pancreatic tumorigenesis, showing both similarities and differences between the two (De Wilde et al., 2012; Iacobuzio-Donahue et al., 2012; Jiao et al., 2011).

A valuable foundation for comprehending tumor heterogeneity was provided by examining the RIP1-TAG2 mouse model as a representation of human PanNETs (Sadanandam et al., 2015). The integration of transcriptomic and metabolic profiling across human and mouse models led to the identification of multiple tumor subtypes, each characterized by unique molecular and clinical features. This work reveals concurrent routes of PanNET carcinogenesis, exhibiting distinctive cells of origin that result in tumor islets and metastasis-like primary subtypes, strengthening the concept of non-linear development of these tumor types.

In conclusion, the approach we introduce offers a foundation for examining variability in the development of diseases, effectively unveiling underlying potential ordinal patterns. Additional research into intricate biological and pathological mechanisms, particularly understanding the distinct developmental routes in both PanNETs and PDAC may have significant implications for prognostic evaluations and tailored treatment plans. While the presented outcomes were obtained from relatively small datasets, further research will focus on external validation with larger sample cohorts, together with the analysis of additional technical modifications. An example could be to complement the OCC sensitivity by alternative OC measures, such as the weighted κ (Cohen, 1968) or Kendall’s τ (Kendall, 1938).

## Data Availability

The original contributions presented in the study are included in the article/supplementary material, further inquiries can be directed to the corresponding author.
